# Survival Time of Visual Gains after Diabetic Vitrectomy and Its Relationship with Ischemic Heart Disease

**DOI:** 10.3390/ijerph17010310

**Published:** 2020-01-02

**Authors:** Abdah Khairiah Che Md Noor, Evelyn Li Min Tai, Yee Cheng Kueh, Ab Hamid Siti-Azrin, Zamri Noordin, Ismail Shatriah

**Affiliations:** 1 Unit of Biostatistics and Research Methodology, School of Medical Sciences, Health Campus, Universiti Sains Malaysia, Kubang Kerian 16150, Malaysia; ab.khairiah@usm.my (A.K.C.M.N.); ctazrin@usm.my (A.H.S.-A.); 2 Department of Ophthalmology, School of Medical Sciences, Health Campus, Universiti Sains Malaysia, Kubang Kerian 16150, Malaysia; evelyn@usm.my (E.L.M.T.); shatriah@usm.my (I.S.); 3 Department of Ophthalmology, Hospital Raja Perempuan Zainab II, Kota Bharu 15586, Kelantan, Malaysia; drzamri.n@moh.gov.my

**Keywords:** type 2 diabetes mellitus, prognostic factors, ischemic heart disease, LogMar, post vitrectomy

## Abstract

Vitrectomy surgery in proliferative diabetic retinopathy improves the vision-related quality of life. However, there is lack of data on the duration of maintenance of visual gains post vitrectomy. This study thus aimed to determine the survival time of visual gains and the prognostic factors of vision loss after vitrectomy surgery for complications of proliferative diabetic retinopathy. A retrospective cohort study was conducted in an ophthalmology clinic in Malaysia. We included 134 patients with type 2 diabetes mellitus on follow-up after vitrectomy for proliferative diabetic retinopathy. Visual acuity was measured using the log of minimum angle of resolution (LogMar). A gain of ≥0.3 LogMar sustained on two subsequent visits was considered evidence of visual improvement post vitrectomy. Subjects were considered to have vision loss when their post-operative visual acuity subsequently dropped by ≥0.3 LogMar. Kaplan–Meier analysis was used to determine the survival time of visual gains. Cox Proportional Hazard regression was used to determine the prognostic factors of vision loss. The median age of patients was 56.00 years (IQR ± 10.00). The median duration of diabetes mellitus was 14.00 years (IQR ± 10.00). Approximately 50% of patients with initial improvement post vitrectomy subsequently experienced vision loss. The survival time, i.e., the median time from surgery until the number of patients with vision loss formed half of the original cohort, was 14.63 months (95% CI: 9.95, 19.32). Ischemic heart disease was a significant prognostic factor of vision loss. Patients with underlying ischemic heart disease (adjusted HR: 1.97, 95% CI: 1.18, 3.33) had a higher risk of vision loss post vitrectomy, after adjusting for other factors. Approximately half the patients with initial visual gains post vitrectomy maintained their vision for at least one year. Ischemic heart disease was a poor prognostic factor for preservation of visual gains post vitrectomy.

## 1. Introduction

As the prevalence of type 2 diabetes mellitus (T2DM) increases, the magnitude of disability secondary to diabetic eye disease-related complications likewise will increase [1]. Worldwide, the estimated overall prevalence of diabetic retinopathy (DR) is 34.6%, while that of vision-threatening diabetic retinopathy is 10.2% [2]. In Kelantan, a state in Malaysia which has one of the highest rates of undiagnosed diabetes, the prevalence of DR has been estimated to be 39.3% [3,4]. In line with the higher prevalence of DR is in developing regions, diabetic patients in emerging nations are especially vulnerable to vision-related functional impairment, especially with more severe grades of retinopathy [5,6]. Recognizing that DR is merely one biomarker of the underlying widespread systemic microvascular effects of abnormal glucose metabolism [7], it is not surprising that patients with DR represent a major public health concern. The health-care costs of patients with DR complications are almost double that of those without the complication, making the societal burden of retinopathy substantial [8].

In patients with proliferative diabetic retinopathy (PDR) complicated by persistent vitreous hemorrhage (VH) or tractional retinal detachment (TRD), pars plana vitrectomy is the only option which offers the potential for patients to regain some degree of their visual function. Unfortunately, although the anatomical results are often excellent, the functional outcome leaves much to be desired [9]. For those patients who do improve post vitrectomy, the gains may be short-lived, due to various causes including recurrent vitreous hemorrhage (VH), cataract, epiretinal membrane (ERM) formation, retinal detachment and neovascular glaucoma [10,11,12,13,14]. This study therefore aimed to estimate the survival time of visual gains and its associated prognostic factors in patients with visual improvement post vitrectomy for complications of PDR.

## 2. Materials and Methods

### 2.1. Study Design and Population

This was a retrospective cohort study involving analysis of medical records of 134 patients who underwent vitrectomy surgery from 1 January 2012 to 31 December 2016 for complications of PDR at Hospital Raja Perempuan Zainab II Kelantan, Malaysia. The patients’ medical records were reviewed until 31 December 2017.

The patients were followed up at the ophthalmology clinic of this hospital. All cases were 20 or 23-gauge primary virectomy with or without tamponade (silicone oil or perfluoropropane gas). Inclusion criteria for the study were: (1) Pseudophakic T2DM patients who underwent primary vitrectomy surgery from 2012 to 2016 for complications of PDR; (2) patients in the group above who experienced visual gains post-surgery (a post-operative gain of ≥0.3 log of minimum angle of resolution (logMAR), sustained on two subsequent visits, was considered evidence of visual improvement); (3) patients on follow up in the ophthalmology clinic after vitrectomy surgery. Exclusion criteria included: (1) Patients with pre-existing concomitant ocular disease e.g., ocular trauma, lens dislocation, macular hole, ERM; (2) incomplete data records for the main variables of interest (more than 30).

### 2.2. Measures

*Outcomes*. The outcome for this study was time to vision loss after vitrectomy surgery and the event of interest was vision loss. The time to vision loss was recorded from day one post vitrectomy until the vision deteriorated during the follow up period. Evaluation of vision loss was conducted by checking the logMAR visual acuity in the patients’ medical records. During the post-operative follow up period, the visual acuity of each patient was measured at day one, week one, week six, month three, month six, and thereafter six-monthly until the last date of follow up. Counting fingers, hand movements, perception of light and no light perception were assigned the values 1.85, 2.3, 2.6, and 2.9 respectively. Subjects were considered to have vision loss when their visual acuity dropped by ≥0.3 LogMar. Censored observations were patients whose visual acuity was maintained until the end of study and those who were lost to follow up. Time for the censored observations was recorded from day one post vitrectomy until the end of the study for patients who maintained their visual acuity, or until the last follow-up date for patients who dropped out from the study.

*Predictors*. Covariates of interest included: socio-demographic characteristics (age; gender; ethnicity; family history of DM); clinical characteristics (duration of DM; anti-diabetic medication); pre-operative features (VH; TRD involving the macula; combined TRD with rhegmatogenous retinal detachment (RRD); laterality; pre-operative iris neovascularization (NVI); pre-operative visual acuity); premorbid conditions (hypertension; ischemic heart disease (IHD); chronic kidney disease; cerebrovascular event); and post-operative complications (post-op VH; post-op TRD; post-op RRD; post-op NVI; post-op ERM).

### 2.3. Data Analysis

The statistical analysis used in this study was survival analysis. In survival analysis, interest centers on a group of individuals for each of whom there is defined point event, often called failure, occurring after a length of time, called failure time, or survival time. A descriptive analysis of socio-demographic, clinical characteristics, premorbid conditions, and post-operative complications was performed using frequency and percentage for categorical variables; while means and standard deviations were used for numerical variables.

Kaplan–Meier analysis was used to determine the survival time of visual acuity among patients with visual improvement post vitrectomy for complications of PDR. The survival time was calculated as the median time taken for survivors (in this case, patients with improved vision) to be equal or less than 50% of the total; i.e., the timing after surgery at which the original cohort of patients who improved was reduced by half. Median times for all categorical independent variables were also estimated using this analysis. The prognostic factors were determined using Cox Proportional Hazard regression. The variables were first analyzed using simple Cox regression. Subsequently, variables with a significant p-value and those which were considered clinically and biologically important were analyzed in multiple Cox regression (variables selection). In multivariable testing, variables with a significant p-value and an alpha error of up to 5% were accepted. Statistical analyses and data entry were performed using IBM SPSS Statistics version 22.0 (IBM Corp, Armonk, NY, USA) [15] and Statistical Data Analysis Software (STATA), version 14.0 (StataCorp LP, College Station, TX, USA) [16].

### 2.4. Ethical Consideration

The study was approved by the Human Research Ethics Committee of Universiti Sains Malaysia [USM/JEPEM/17070326] and was registered under the National Medical Research Registry, Ministry of Health Malaysia. The conduct of the study followed the tenets of the declaration of Helsinki.

## 3. Results

### 3.1. Overall Characteristics

Table 1 shows the demographic and clinical features of our study subjects. There were 134 patients who met the inclusion and exclusion criteria. In total, 67 (50%) vision losses were reported, while the remaining 67 (50%) were censored observations. Among the censored group, 18 cases were due to loss to follow up (27%). The gender distribution was approximately equal, with 67 (50%) males and 67 (50%) females. The median age was 56.00 years (IQR ± 10.00) and the median duration of DM was 14.00 years (IQR ± 10.00).

**Figure 1:**
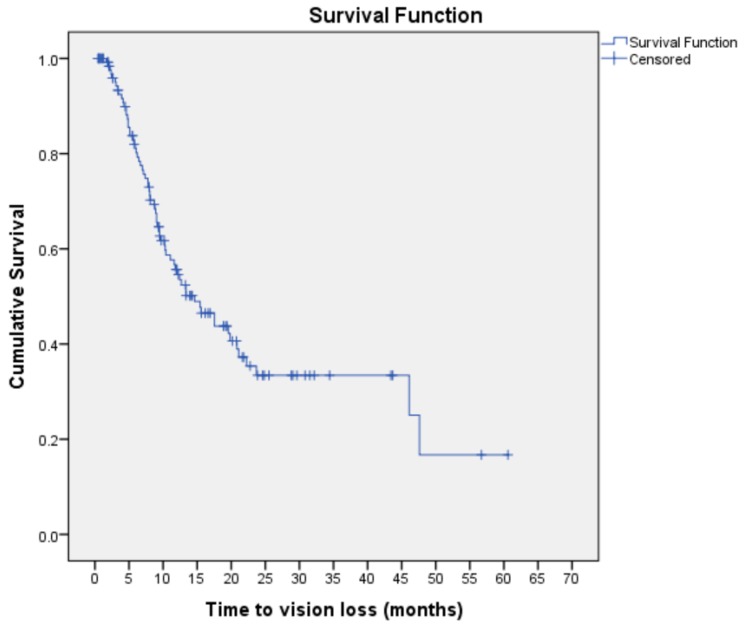
Kaplan–Meier curve for vision loss estimates among subjects with visual improvement post vitrectomy for complications of PDR (n = 134).

The majority of patients (91.0%) had hypertension, while about a third (32.8%) had chronic kidney disease and almost a quarter (26.1%) had IHD. Pre-operatively, 77 patients (57.5%) had TRD with macula involvement. Post-vitrectomy, half of this cohort experienced VH, while another one-third had TRD. Based on Kaplan–Meier analysis, the overall median time to vision loss was 14.63 months (95% CI: 9.95, 19.32) (Figure 1). Figure 1 shows the median probability time of 14.63 months, i.e., the estimated median time at which 50% of the patients had vision loss. Figure 1 provides a visual illustration of the probability of vision loss at a given time interval (i.e., month).

The Kaplan–Meier curve shows the time to vision loss for individual study subjects throughout the duration of the study. The horizontal lines along the X-axis represent the survival duration in months, i.e., the duration until vision loss occurs. All subjects begin the analysis at the same point and are considered ‘survivors’ until an event of interest (vision loss) occurs. Each event is marked by a sharp downward deflection of the graph (vertical line). Censored patients (i.e., subject maintaining vision or lost to follow up) are also marked on the graph.

### 3.2. Unadjusted Factors of Vision Loss

Using univariable analysis of simple Cox regression, only one variable, IHD, was statistically significant (*p*-value = 0.009). Five other factors with a *p*-value less than 0.25 were duration of DM (*p*-value = 0.230), TRD with macula involvement (*p*-value = 0.134), post-operative VH (*p*-value = 0.102), post-operative TRD (*p*-value = 0.095) and post-operative ERM (*p*-value = 0.171) (Table 2).

### 3.3. Prognostic Factors of Vision Loss

After multivariable analysis evaluating for age, duration of DM, gender, pre-operative VH, pre-operative TRD with macula off, pre-operative combined TRD/RRD, CKD, pre-operative NVI, IHD, post-operative TRD, post-operative VH and post-operative ERM, we found that only the variable IHD (*p*-value = 0.010) was significantly associated with vision loss. The preliminary main effect model was achieved.

## 4. Discussion

Vitrectomy surgery has been observed to improve the vision-related quality of life in patients with PDR, likely due to its association with visual gains [17]. However, most studies assessing the functional effect of vitrectomy in DR fail to discuss the time-limited nature of these gains [17,18]. To the best of our knowledge, this study is the first to identify the survival time of visual gains among patients with visual improvement post vitrectomy for complications PDR. We also evaluate the factors associated with vision loss in this cohort.

We found that 50% of our cohort experienced visual loss during the study period. This may seem to compare unfavorably with other published results of vitrectomy [11,19], but differences in study objective may serve to explain the discrepancy. Most studies evaluating the outcome of vitrectomy measure success based on visual acuity at a single point in time, and compare their results of patients with improved vision to those who experienced no improvement with surgery. In contrast, the focus of our study was only on those who improved with surgery; we were interested in the duration of time that these patients could hope to enjoy their newly regained vision. Our 14.63 months survival time of visual gains post vitrectomy reflects the median time from surgery until 50% of our cohort sustained visual loss. These results may be useful to temper patient expectations during counseling for surgery. However, considering the life expectancy of diabetic patients requiring vitrectomy and their 5-year survival rates, which may be as low as 68%, the decision for vitrectomy may still be seen as a worthwhile investment to optimize the quality of their remaining years of life [20,21,22,23,24].

We observed that the variable IHD was significantly associated with vision loss among patients who underwent vitrectomy for complications of PDR. To the best of our knowledge, our study is the first to identify IHD as a prognostic factor for vision loss among PDR patients. IHD has previously been identified as a poor prognostic factor for survival after diabetic vitrectomy, with 50% of these patients dying within 3.5 years of surgery [25]. Cardiovascular disease is in fact the most common cause of death in patients undergoing vitrectomy for diabetic retinopathy [24]. We believe that these results reflect the underlying insufficiency of the vasculature in diabetic patients. Diabetic retinopathy is associated with micro- and macrovascular complications of diabetes, and its progression increases the risk of stroke and cardiovascular disease [26]. T2DM patients with PDR have been observed to have a higher risk of cardiovascular disease [27,28] than those without PDR. This association is supported by studies demonstrating that retinopathy is associated with a higher risk of abnormal perfusion [29], possibly related to impaired arteriolar dilation in response to hypoxia [30]. As no overt associations such as VH, TRD and ERM were found in our patients who experienced vision loss, we hypothesize that the cause for their visual deterioration is related to retinal ischemia. Although differences in the pathophysiology of macro and microvascular disease are beyond the scope of this discussion, both retinopathy and coronary artery disease share a common end pathway of endothelial dysfunction. This may explain the observed benefit of statins in these conditions, which have been shown to have immune-modulatory effects on inflammation [31,32] and endothelial dysfunction [33,34,35,36].

Post-operative VH and TRD were not uncommon in our cohort, and reflect the published rates of these complications [10,37]. However, they did not have a statistically significant effect on maintenance of visual gains post vitrectomy. Our study also found that other factors traditionally associated with a poor visual outcome post diabetic vitrectomy (such as pre-operative NVI [38,39], and TRD [11]) were not significantly associated with vision loss. This is due to difference in study design, as discussed above; most studies of diabetic vitrectomy evaluated overall visual outcome in all vitrectomy cases, while we more concerned with maintenance of visual gains within the group of patients who experienced visual improvement post vitrectomy. In the former, pre-operative conditions like long-standing macular detachment may have limited visual gains. In the latter, as discussed above, systemic risk factors may play a greater role than previously acknowledged, because although post-operative ocular complications like recurrent VH may be managed with a repeat vitrectomy, the underlying vascular dysfunction in these patients remain to be addressed.

Our study is not without its limitations, such as selection bias, which may have resulted in sampling a greater number of subjects with visual loss post-vitrectomy as those with good vision may have been tempted to default follow-up. Secondly, although patients were assumed to have macular ischemia in the absence of any overt causes of poor final visual acuity, objective documentation such as via fundus fluorescein angiography was generally not available. Finally, due to the retrospective nature of this study, the diagnosis of IHD was loosely defined, and further relevant information such as medication and smoking history could not be ascertained. Thus, the possibility remains that these and other unmeasured confounders may explain the observed association. Prospective designs with special attention to evaluation of cardiovascular risk factors, lifestyle and medication history may address these issues, although loss to follow-up remains a valid concern, especially over long periods of time.

The perceived value of sight can perhaps best be illustrated by the results of online surveys conducted among the general public, in which loss of sight is rated as the worst possible health outcome [40,41]. On average, respondents chose 4.6 years of perfect health over 10 years of life with complete sight loss [41]. The unique strength of our study is thus its identification of the survival time of vitrectomy-related visual gains, which will not only assist clinicians in providing patients with realistic expectations of their post-surgical outcome, but also guide the development of timely post-operative review schedules. Knowledge of the risk factors for vision loss also allows identification of high-risk patients. Future research involving risk factor modification in this group may shed further information on the significance of these findings.

## 5. Conclusions

Approximately 50% of patients with initial post vitrectomy visual improvement will maintain their vision for at least 14 months after surgery. The hazards of visual loss are higher in those with pre-existing IHD.

## Figures and Tables

**Table 1:** Characteristics of subjects with visual improvement post vitrectomy for complications of PDR (n = 134).

Characteristics	Vision Loss	Censored	Total
	Frequency (%)	Frequency (%)	Frequency (%)
Gender			
Male *	37 (55.2)	30 (44.8)	67 (50.0)
Female	30 (44.8)	37 (55.2)	67 (50.0)
Ethnicity			
Non-Malay *	5 (7.5)	8 (11.9)	13 (9.7)
Malay	62 (92.5)	59 (88.1)	121 (90.3)
Family history of DM			
Yes	67 (100)	67 (100)	134 (100)
Type of DM			
Type II	67 (100)	67 (100)	134 (100)
Anti-diabetic medication			
Oral medication *	12 (17.9)	13 (19.4)	25 (18.7)
Insulin	55 (82.1)	54 (80.6)	109 (81.3)
Laterality			
Right eye *	28 (41.8)	31 (46.3)	59 (44.0)
Left eye	39 (58.2)	36 (53.7)	75 (56.0)
Pre-operative VH			
Yes	55 (82.1)	55 (82.1)	110 (82.1)
No *	12 (17.9)	12 (17.9)	24 (17.9)
Pre-operative TRD with macula involvement			
Yes	37 (55.2)	40 (59.7)	77 (57.5)
No *	30 (44.8)	27 (40.3)	57 (42.5)
Pre-operative combined TRD/RRD			
Yes	9 (13.4)	5 (7.5)	14 (10.5)
No *	58 (86.6)	62 (92.5)	120 (89.5)
Pre-operative NVI			
Yes	3 (4.5)	2 (3.0)	5 (3.7)
No *	64 (95.5)	65 (97.0)	129 (96.3)
Hypertension			
Yes	63 (94.0)	59 (88.1)	122 (91.0)
No *	4 (6.0)	8 (11.9)	12 (9.0)
IHD			
Yes	22 (32.8)	13 (19.4)	35 (26.1)
No *	45 (67.2)	54 (80.6)	99 (73.9)
CKD			
Yes	26 (38.8)	18 (26.8)	44 (32.8)
No *	41 (61.2)	49 (73.1)	90 (67.2)
CVA			
Yes	1 (1.5)	0 (0)	1 (0.8)
No *	66 (985)	67(100)	133 (99.2)
Post-Op VH			
Yes	40 (59.7)	27 (40.3)	67 (50.0)
No *	27 (40.3)	40 (59.7)	67 (50.0)
Post-Op TRD			
Yes	24 (35.8)	19 (28.4)	43 (32.1)
No *	43 (64.2)	48 (71.6)	91 (67.9)
Post-Op RRD			
Yes	5 (7.5)	2 (3.0)	7 (5.2)
No *	62 (92.5)	65 (97.0)	127 (94.8)
Post-op NVI			
Yes	8 (11.9)	3 (4.5)	11 (8.2)
No *	59 (88.1)	64 (95.5)	123 (91.8)
Post-op ERM			
Yes	13 (19.4)	11 (16.4)	24 (17.9)
No *	54 (80.6)	56 (83.6)	110 (82.1)

Note. DM: Diabetes mellitus, VH: Vitreous hemorrhage, TRD: Tractional retinal detachment, RRD: Rhegmatogeneous retinal detachment, NVI: Neovascularization of iris, IHD: Ischemic heart disease, CKD: Chronic kidney disease, CVA: Cerebrovascular accident, ERM: Epiretinal membrane; * Reference group.

**Table 2:** Prognostic factors associated with vision loss among subjects with visual improvement post vitrectomy for complications of proliferative diabetic retinopathy (Simple Cox regression) (n = 134).

Variables	Regression Coefficient (b)	Crude Hazard Ratio (95% CI) ^1^	Wald Statistic	*p*-Value
Age	0.02	1.02 (0.98, 1.06)	0.28	0.279
Gender				
Male *	0	1.00	-	-
Female	0.01	1.10 (0.63, 1.64)	0.95	0.954
Ethnicity				
Non-Malay *	0	1.00	-	-
Malay	0.33	1.39 (0.56, 3.47)	0.46	0.477
Duration of DM	0.02	1.02 (0.99, 1.06)	0.24	0.230
Anti-diabetic medication				
Oral medication *	0	1.00	-	-
Insulin	0.27	1.31 (0.70, 2.46)	0.39	0.398
Laterality				
Right eye *	0	1.00	-	-
Left eye	−0.01	1.00 (0.61, 1.63)	1.00	0.998
Pre-operative VH				
Yes	−0.35	0.70 (0.37, 1.31)	1.29	0.269
No *	0	1.00	-	-
Pre-operative TRD with macula involvement				
Yes	0.38	1.47 (0.89, 2.43)	0.13	0.134
No *	0	1.00	-	-
Pre-operative combined TRD/RRD				
Yes	0.10	1.11 (0.54, 2.24)	0.78	0.782
No *	0	1.00	-	-
Pre-operative NVI				
Yes	0.56	1.75 (.55, 5.g)	0.38	0.344
No *	0	1.00	-	-
IHD				
Yes	0.69	1.99 (1.18, 3.35)	0.01	0.009
No *	0	1.00	-	-
CKD				
Yes	0.11	1.11 (0.68, 1.82)	0.67	0.668
No *	0	1.00	-	-
Post-Op VH				
Yes	0.41	1.50 (0.92, 2.45)	0.10	0.102
No *	0	1.00	-	-
Post-Op TRD				
Yes	0.43	1.54 (0.93, 2.56)	0.10	0.095
No *	0	1.00	-	-
Post-op ERM				
Yes	−0.43	0.65 (0.35, 1.20)	0.15	0.171
No *	0	1.00	-	-

Note. 95% CI = confidence interval; ^1^ Simple Cox proportional hazard regression; DM: Diabetes mellitus, VH: Vitreous hemorrhage, TRD: Tractional retinal detachment, RRD: Rhegmatogeneous retinal detachment, NVI: Neovascularization of iris, IHD: Ischemic heart disease, CKD: Chronic kidney disease, ERM: Epiretinal membrane; * Reference group.
